# Disease Surveillance and the Academic, Clinical, and Public Health Communities

**DOI:** 10.3201/eid0907.030083

**Published:** 2003-07

**Authors:** Robert W. Pinner, Catherine A. Rebmann, Anne Schuchat, James M. Hughes

**Affiliations:** * Centers for Disease Control and Prevention, Atlanta, Georgia, USA

**Keywords:** communicable diseases, emerging, pneumococcal vaccines, streptococcal infections, food poisoning, syndrome, bioterrorism, Centers for Disease Control and Prevention, population surveillance, public health, disease notification, Synopsis

## Abstract

The Emerging Infections Programs (EIPs), a population-based network involving 10 state health departments and the Centers for Disease Control and Prevention, complement and support local, regional, and national surveillance and research efforts. EIPs depend on collaboration between public health agencies and clinical and academic institutions to perform active, population-based surveillance for infectious diseases; conduct applied epidemiologic and laboratory research; implement and evaluate pilot prevention and intervention projects; and provide capacity for flexible public health response. Recent EIP work has included monitoring the impact of a new conjugate vaccine on the epidemiology of invasive pneumococcal disease, providing the evidence base used to derive new recommendations to prevent neonatal group B streptococcal disease, measuring the impact of foodborne diseases in the United States, and developing a systematic, integrated laboratory and epidemiologic method for syndrome-based surveillance.

During the 1980s, clinicians added newly recognized infectious diseases, such as toxic shock syndrome and AIDS, to their differential diagnoses when evaluating previously healthy young adults with severe illness. More recently, clinicians in the United States found themselves considering the possibility of inhalational anthrax among patients with influenzalike illnesses and adding West Nile virus infection to their workup of posttransfusion fevers (*1*–*3*). The existence of these and dozens of other emerging and reemerging infectious diseases, naturally or intentionally transmitted, has removed any doubt about the interdependence of clinical medicine and public health. Clinicians are sentinels for detection of new or reemerging diseases and may benefit from information acquired through public health surveillance and research projects, which helps to place the quantitative risks of these new diseases in perspective amidst the media attention that often accompanies the latest medical mysteries.

In 1992, the Institute of Medicine (IOM) articulated the concept of emerging infections, discarding the naive view that infectious diseases were problems of the past and cautioning against complacency about public health preparedness for infectious diseases (*4*). By defining emerging infectious diseases as “new, reemerging, or drug-resistant infections whose incidence in humans has increased within the past two decades or whose incidence threatens to increase in the near future,” IOM recognized the broad scope of these diseases. The IOM report also cited factors that influence the emergence of infectious diseases: changes in human demographics and behavior; advances in technology and changes in industry practices; economic development and change in land-use patterns; increased volume and speed of international travel and commerce; microbial adaptation and change; and breakdown of public health capacity at the local, national, and global levels. The intentional release of anthrax in the United States in 2001 emphasized the need to add intentionally inflicted harm to the list of factors that influence the emergence of infectious diseases and to suspect the unexpected.

In response to the IOM report, Addressing Emerging Infectious Disease Threats to Health: A Prevention Strategy for the United States was developed by the Centers for Disease Control and Prevention (CDC) (*5*). A key recommendation of the plan called for establishing population-based centers to complement and support local, regional, and national surveillance and research efforts. This concept was realized through Emerging Infections Programs (EIPs), a network of state health departments (Figure 1) coordinated by CDC. EIPs are intended to be a national resource for surveillance and epidemiologic research by conducting work that goes beyond the routine public health department functions; by fostering collaborations between the public health, academic, and clinical communities; and by maintaining an infrastructure flexible enough to address new infectious diseases challenges as they emerge. An updated plan released in 1998 described the important role assumed by EIPs in addressing emerging infections and identified several high-priority target areas (*6*), which include: antimicrobial drug resistance, foodborne and waterborne diseases, vector-borne and zoonotic diseases, chronic diseases caused by infectious agents, diseases transmitted through blood transfusions or products, vaccine development and use, diseases of pregnant women and newborns, diseases of persons with impaired host defenses, and diseases of travelers, immigrants, and refugees. We describe EIP accomplishments and future directions.

**Figure 1:**
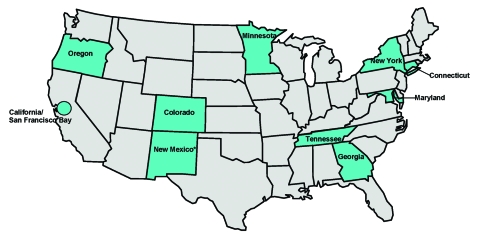
Distribution of Emerging Infections Programs (EIPs), a network of 10 state health departments and their collaborators in local health departments, academic institutions, and clinical settings, coordinated by the Centers for Disease Control and Prevention. *New Mexico was added as the 10th EIP site in late 2002 and will begin EIP activities during 2003.

## EIP Methods

The principal functions of EIPs are to perform active, population-based surveillance for infectious diseases; conduct applied epidemiologic and laboratory research; implement and evaluate pilot prevention and intervention projects; and provide capacity for flexible public health response. EIPs also develop and evaluate public health practice and transfer what is learned to the public health and medical communities.

These programs are supported through cooperative agreements between CDC and state health departments, who engage collaborators in local health departments, hospitals, and academic institutions. Additional funding for certain EIP activities comes from other sources; for example, the U.S. Department of Agriculture and the Food and Drug Administration provide support for activities involving foodborne illnesses, and the National Vaccine Program Office has provided support for postlicensure vaccine evaluations.

The population base for EIP activities is approximately 36 million persons, though the base varies by project. This population represents an approximation of the U.S. population with respect to demographic characteristics such as age, gender, race, and urban residence, as well as health indicators such as population density and percentage of persons at or below the poverty level (*7*). EIPs are geographically dispersed throughout the country (Figure 1).

Active, laboratory-based surveillance is the foundation of two core EIP projects conducted at all sites: Active Bacterial Core Surveillance (ABCs) and Foodborne Disease Active Surveillance (FoodNet) (Table 1). These active surveillance projects generate reliable estimates of the incidence of certain infections and provide the foundation for a variety of epidemiologic studies to explore risk factors, disease spectrum, and prevention strategies (*8*,*9*). For example, the total impact of foodborne illnesses in the United States has been estimated by combining FoodNet active surveillance data with other data sources and results from FoodNet surveys of the general population (to learn about the frequency of diarrhea in the general population and to determine what proportion of persons with diarrhea seeks medical care), physicians (to determine the frequency of stool-culturing by physicians), and clinical laboratories (to determine the frequency of culturing for selected foodborne pathogens) (*9*–*11*). These data provide estimates of the overall occurrence of diarrheal illness (0.7 illnesses/person-year), as well as the likely degree of underreporting for specific infections under surveillance (*10*).

**Table 1:** Surveillance and focus area for two core projects conducted at all Emerging Infections Program sites^a^

Projects	Type of surveillance	Focus
Active Bacterial Core Surveillance	Active, laboratory-based	Invasive disease (isolated from a normally sterile site such as blood or cerebrospinal fluid) caused by group A streptococcus, group B streptococcus, *Haemophilus influenzae, Neisseria meningitidis,* and *Streptococcus pneumoniae*
FoodNet/Foodborne Disease Active Surveillance	Active, laboratory-based	Disease (first isolation from a person) caused by *Campylobacter, Listeria, Salmonella, Shigella, Yersinia, Vibrio,* Shiga toxin–producing *Escherichia coli*, including O157:H7, *Cryptosporidium,* and *Cyclospora*

^a^Intended to generate reliable estimates of the incidence of certain infections and provide the foundation for a variety of epidemiologic studies to explore risk factors, disease spectrum, and prevention strategies.

Other projects are conducted by EIPs, depending on local priorities and expertise. The Unexplained Deaths and Critical Illness (UNEX) project, a prospective study that uses epidemiologic and laboratory methods to detect and investigate unexplained illnesses with clinical features suggesting infectious diseases, has been in place at four states with EIPs since the inception of the program (*12*,*13*). The Connecticut EIP conducts active surveillance for emerging tick-borne diseases that are transmitted by a single tick vector (*Ixodes scapularis*) in the state (*14*). EIPs also strive to maintain the flexibility to meet new challenges effectively. For example, in 1996 four EIP sites conducted active surveillance for variant Creutzfeldt Jacob Disease (CJD) and physician-diagnosed CJD cases. This study contributed to surveillance methods by confirming that death certificate reviews are a sensitive method for detecting CJD deaths while providing some assurance that variant CJD was not occurring in these states (*15*).

## Impact of a New Pneumococcal Vaccine

Through ABCs, we are evaluating the effect of the pneumococcal conjugate vaccine on the epidemiology of invasive pneumococcal disease in the United States. *Streptococcus pneumoniae* (pneumococcus), which is an important cause of serious illness among young children, is the leading cause of bacterial pneumonia and meningitis in the United States. For many years, immunization against pneumococcus with a 23-valent polysaccharide vaccine was recommended for persons >2 years of age who are at high risk and for all adults >65 years of age. Although disease incidence is highest in the first 2 years of life, the polysaccharide vaccine was poorly immunogenic in this group. In February 2000, a protein-polysaccharide pneumococcal conjugate vaccine for seven pneumococcal serotypes (Prevnar, Wyeth Pharmaceuticals, Pearl River, NY) was licensed for use in infants and children (*16*). This conjugate vaccine is now recommended in the United States for all children <2 years of age, with catch-up vaccination schedules suggested for children 2 to 4 years of age. In clinical trials, the vaccine was efficacious against invasive disease in infancy and reduced nasopharyngeal colonization by vaccine-type strains, an indication of potential for herd immunity.

One method used by ABCs is to collect available isolates from identified cases. Serotyping data were analyzed to learn about the epidemiology of *S.* *pneumoniae* in the pre-conjugate vaccine era and to predict the potential impact of the conjugate vaccine (*17*). Of pneumococcal cases identified by ABCs from 1995 to 1998, at least 82% in children <2 years of age were caused by serotypes included in the 7-valent pneumococcal conjugate vaccine. These population-based ABCs data were used to formulate the original pneumococcal conjugate vaccine schedules and provide recommendations for administering the vaccine to infants and children. When a vaccine shortage became evident in 2001, ABCs data were again used by public health officials to weigh alternative strategies for delivering available doses (*18*). Surveillance is now focused on evaluating changes in disease impact after the conjugate vaccine was introduced, including whether it interrupts transmission of antibiotic-resistant pneumococci. Analysis of ABCs data shows a substantial decline in disease caused by serotypes in the vaccine formulation among children in the age group for whom the vaccine is recommended. More modest declines also occur in selected adult groups (*19*).

ABCs will continue to evaluate the impact of the recently introduced pneumococcal conjugate vaccine, including whether vaccine shortages have slowed the initial steep decline in disease occurrence. Other goals are measurement of vaccine efficacy, assessment of whether the vaccine is interrupting transmission, and evaluation of the distribution of serotypes causing disease (to determine if decline in disease because of serotypes included in the vaccine has been counterbalanced by emergence of invasive disease caused by nonvaccine serotypes). While this “replacement disease” phenomenon was recognized for otitis media and colonization in the prelicensure vaccine trials, no evidence of replacement invasive disease has thus far been recognized.

Clinicians were challenged by the emergence of multidrug-resistant pneumococci during the 1990s, when new treatment guidelines were developed for meningitis, otitis media, and pneumonia (*20*). Vaccines, in concert with campaigns to promote appropriate use of antibiotics, provide opportunities to transform the problem of drug-resistant pneumococci from a treatment dilemma to a prevention success story (*21*).

## Revised Recommendations for Preventing Perinatal Group B Streptococcal Disease

**Figure 2:**
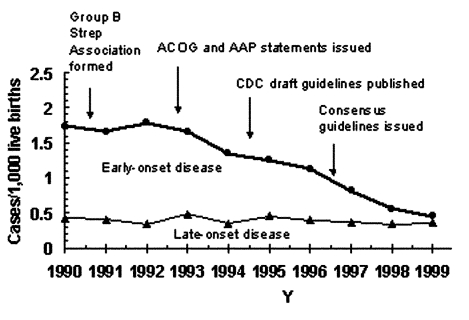
Incidence of early- and late-onset invasive group B streptococcal disease in three active surveillance areas (California, Georgia, and Tennessee), 1990–1998, and activities for the prevention of group B streptococcal disease (*22*). CDC, Centers for Disease Control and Prevention. ACOG, American College of Obstetricians; AAP, American Academy of Pediatrics.

Data developed through ABCs provided a basis for revising recommendations for the prevention of perinatal group B streptococcal (GBS) disease. Since its emergence in the 1970s, GBS disease has been the leading invasive bacterial infection associated with illness and death among newborns in the United States. Surviving infants may have long-term developmental disabilities, such as mental retardation or hearing and vision loss. Newborns at increased risk for GBS disease are those born to women who are colonized with GBS in the genital or rectal areas. Although the use of intrapartum prophylaxis has led to a 70% decline in the incidence of GBS disease during the 1990s (Figure 2) (*22*,*23*), early-onset GBS disease (in infants <7 days old) remains a leading cause of illness and death among newborns. Guidelines issued in 1996 recommended either screening pregnant women for GBS colonization by means of prenatal cultures (screening approach) or assessing obstetric risk factors intrapartum (risk-based approach) to identify candidates for intrapartum antibiotic prophylaxis.

An EIP population-based, retrospective cohort study compared the effectiveness of prenatal screening for GBS with the risk-based approach for preventing early-onset GBS sepsis (*24*). The analysis, which combined ABCs population-based active surveillance data on GBS cases with a sample survey representing >600,000 deliveries, showed that infants born to women who had been screened for GBS before delivering had less than half the risk for early-onset GBS compared to infants of women who had not been screened, after adjustments were made for potential confounders. The protective effect of the screening approach resulted mainly from broader coverage of the population at risk because many early-onset GBS cases in the preprevention era occurred in GBS-colonized women without obstetric risk factors. The evidence for updated prevention recommendations from key health organizations (i.e., American College of Obstetricians and Gynecologists, American Academy of Pediatrics, American College of Nurse-Midwives, and CDC) was based on the finding that routine screening for GBS during pregnancy more effectively prevents cases of early-onset disease than the risk-based approach (*25*). Through ABCs, CDC will continue to monitor GBS disease trends to understand the impact of the new recommendations and detect potential adverse consequences of intrapartum antibiotic use such as emergence of sepsis caused by other organisms or new patterns of antimicrobial resistance (*26*,*27*).

## Decrease in Bacterial Foodborne Diseases

FoodNet documented a decrease in bacterial foodborne illnesses from 1996 to 2001. Many infections are transmitted through food and can cause illness ranging from mild gastroenteritis to severe illness requiring hospitalization. Foodborne pathogens cause an estimated 76 million illnesses, 325,000 hospitalizations, and 5,000 deaths in the United States each year (*11*). Clinicians treating patients with acute gastroenteritis are principally focused on whether empiric antimicrobial agents are warranted and the value of diagnostic evaluation. However, the task of providing accurate information on trends in specific foodborne pathogens capable of causing this syndrome, as well as probable sources of infection, has historically fallen to public health authorities.

**Figure 3:**
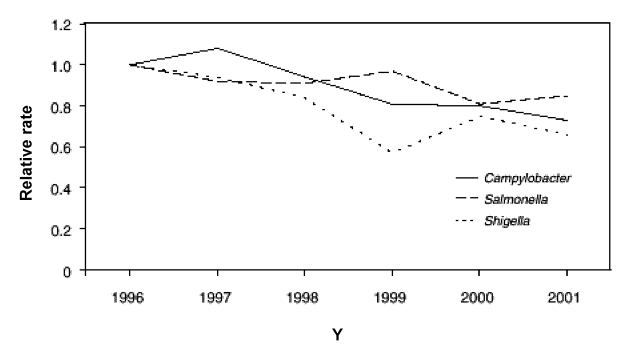
Relative rates compared with 1996, adjusted for sites, of laboratory-diagnosed cases of *Campylobacter*, *Salmonella*, and *Shigella*, by year, FoodNet, United States, 1996–2001 (*28*). Bacterial pathogens with highest incidences of the 10 studied diseases are shown.

Data from FoodNet documented recent declines in the occurrence of several major bacterial foodborne illnesses (*9*,*28*); preliminary surveillance data for 2001 were compared with 1996–2000 data (*28*). Significant declines occurred in major bacterial foodborne illnesses, including infections caused by *Yersinia* (49%), *Listeria* (35%), *Campylobacter* (27%), and *Salmonella* (15%) (Figure 3). The combined estimated incidence of infections caused by *Listeria*, *Campylobacter*, *Salmonella*, and *E. coli* O157 in 2001 was 21% lower than in 1996, on the basis of a multivariate regression model.

The factors influencing the occurrence of foodborne illnesses are complex. However, the observed declines in foodborne disease incidence did occur in the context of several control measures, including the U.S. Department of Agriculture’s Food Safety Inspection Service’s implementation of the Pathogen Reduction/Hazard Analysis and Critical Control Point regulations in meat and poultry slaughter and processing plants, egg-quality assurance programs for *Salmonella* Enteritidis, and increased consumer education in food safety (*28*).

FoodNet will continue to monitor the occurrence of foodborne diseases. In 2003, FoodNet will also conduct studies of the consequences of and risk factors for illness caused by *S.* Enteritidis, *S.* Newport, and illness in infants caused by *Campylobacter* and *Salmonella*. Other activities include a project to improve collection and transport of specimens during outbreaks so that a cause is identified in a higher percentage of outbreaks.

Rapid identification of a cause for cases of infectious diarrhea and appropriate reporting of cases of foodborne illnesses to state or local public health authorities are important not only in identifying and controlling outbreaks but also for more precise assessments of the local, regional, and national trends in foodborne illnesses (*29*). In turn, such estimates can inform clinicians of likely causes, probable sources, and prognostic factors for episodes of illness in persons under their care.

## Unexplained Deaths and Critical Illnesses Project

Many clinicians have treated patients with puzzling situations, in which the acute onset of a critical illness suggestive of an infectious origin occurred in otherwise healthy young people for whom diagnostic tests failed to identify an etiologic agent. Occasionally, such episodes are retrospectively diagnosed many years later with the recognition of a new infectious disease and testing of stored clinical specimens. For example, hantavirus pulmonary syndrome was first recognized and described in the United States in 1993 by an alert clinician during an outbreak in the Southwest (*30*); retrospective reviews of fatal illnesses showed that unrecognized cases of hantavirus pulmonary syndrome had preceded the 1993 outbreak by at least 15 years (*31*). Similarly, cases of legionellosis and AIDS were recognized in hindsight years after they had occurred (*13*). These observations, coupled with the new laboratory techniques for pathogen identification, particularly methods that do not rely on culture, suggested that an effort to prospectively identify pathogens causing unexplained syndromes might yield useful information (*12*,*13*); this was the beginning of the UNEX project. Laboratory evaluation of cases includes traditional serologic and in vitro culture diagnostic methods as well as molecular techniques. This combined epidemiologic and laboratory approach is a hallmark feature of other EIP projects that study hepatitis, acute respiratory diseases, and encephalitis (*32*).

The UNEX project has developed methods for evaluating severe syndromes indicating infection, including nonculture-based methods to identify etiologic agents. From May 1, 1995, to December 31, 1998, 137 illnesses meeting the UNEX case definition were reported to participating EIPs. After adjustments for age and race were made, this number translates to an estimated 920 U.S. cases per year; the overall annual incidence rates did not change during this time. No differences were observed in the seasonal distribution of cases of unexplained illnesses, nor did cases cluster by time or place. The largest proportion of cases was treated as a neurologic syndrome (29%), followed by respiratory (27%) and cardiac (21%) syndromes. Diagnostic testing through UNEX identified a cause in 34 (28%) of 122 cases from which specimens were available (Table 2).

**Table 2:** Infectious causes and explanations for unexplained deaths and critical illnesses cases, 1995–1998, California, Oregon, Connecticut, and Minnesota (n=34)^a,b^

Syndrome	Etiology (n)	Tests (n)
Neurologic (n=15)	*Neisseria meningitidis* (4)	16S rDNA PCR (2), PCR (1), EIA IgM (1)^a^
	*Bartonella hensaelae* (1)	PCR, IFA, IgG
	*Bartonella* spp. (2)	IFA, IgG
	*Chlamydia pneumoniae* (1)	MIF, IgG
	*Mycoplasma pneumoniae* (1)	EIA, IgM/IgG
	*Cytomegalovirus* (1)	EIA and IFA,IgG
	Coxsackie B (1)	EIA,IgM, viral culture
	Enterovirus (1)	EIA,IgM
	Epstein-Barr virus (1)	IFA,IgG (VCA and EA)
	Human herpes virus 6 (1)	IFA and EIA (IgM and IgG)
	Mumps virus (1)	IFA IgM, IFA and EIA, IgG
Respiratory (n=13)	*Chlamydia pneumoniae* (2)	MIF IgG (2), IFA, IgM
	*Mycoplasma pneumoniae* (4)	PCR (blood), EIA, IgM/IgG
	*Streptococcus pneumoniae* (2)	16S rDNA PCR (pleural fluid)
	*Legionella* spp. (1)	PCR (from lung)
	Adenovirus (1)	EIA and IFA, IgG
	Influenza B virus (1)	EIA and IFA, IgG
	Influenza A virus (1)	EIA and IFA, IgM, EIA (IgG)
	Human parainfluenza virus types 1 and 3 (1)	EIA and IFA, IgG
Cardiac (n=3)	*Borrelia burgdorferi*/*Ehrlichia chaffeensis* (1)	EIA/IFA flagella, IgG, Western Blot (IgG and IgM)
	Enterovirus (1)	EIA IgM
	*Legionella* spp. (1)	PCR (heart)
Multisystem (n=3)	*Neisseria meningitidis* (1)	PCR (cerebrospinal fluid)
	Adenovirus (1)	PCR (blood)
	Enterovirus (1)	IgM, EIA

^a^ PCR, polymerase chain reaction; EIA, enzyme immunosorbent assay; IFA, indirect immunofluorescent assay; IG, immunoglobulin; MIF, microimmunofluorescence.

^b^Reference *12*.

Two recent outbreaks demonstrate the usefulness of the approach developed for UNEX. During a 1999 outbreak of West Nile encephalitis in the northeastern United States, which was recognized by an alert clinician (*33*), and during an outbreak of unexplained illness among injecting drug users in Scotland and Ireland (*34*), initial reports of illness were received and initial laboratory testing performed through the laboratory infrastructure established for the UNEX project.

The frequency and distribution of the syndromes identified through this project undoubtedly reflect both the distribution of their occurrence and gaps in our ability to diagnose causes of neurologic and respiratory syndromes in particular. Although novel pathogens have not yet been discovered through the UNEX project, this systematic approach improves chances of recognizing infectious disease causes earlier than in the past and lays the groundwork for the development of improved diagnostic tools. Moreover, concerns about bioterrorism have put a premium on the early detection of an intentional release or infectious or chemical agents; this syndrome-based surveillance, which seeks early identification and diagnosis, can contribute to public health preparedness for such events.

## Future Directions of EIPs

Since the release of the plan that launched the EIPs, these programs have made substantial contributions to the practice of U.S. public health. Using domestic EIPs as a model, CDC has begun developing a network of international EIPs (IEIPs) in collaboration with Ministries of Health and other international partners. The first IEIP was established in Thailand during 2001, and a second IEIP is being established in Kenya. Collaborations between EIPs and IEIPs will provide valuable opportunities for training. In addition, the new U.S. EIP in New Mexico will feature work along the U.S.-Mexico border and also promises to enhance international collaborations.

Opportunities presented by new laboratory and information technologies, as well as challenges posed by potential bioterrorism, will influence the evolution of the EIPs over the next several years. EIP work will build on experience gained through the combined epidemiologic and laboratory evaluation of syndromes to enhance bioterrorism preparedness and develop the capacity for identifying previously unrecognized pathogens. However, even as new technologies are found, knowledgeable and engaged clinicians will remain a vital element in efforts to detect, respond to, and prevent emerging infectious diseases.
